# Endoscopic submucosal dissection for a duodenal polyp at the upper aspect of the duodenal bulb using a newly developed endoscope

**DOI:** 10.1016/j.vgie.2023.07.016

**Published:** 2023-09-12

**Authors:** Satoki Shichijo, Yushi Kawakami, Atusko Kizawa, Daiki Kitagawa, Yasuhiro Tani, Yoji Takeuchi, Ryu Ishihara

**Affiliations:** Department of Gastrointestinal Oncology, Osaka International Cancer Institute, Osaka, Japan

Endoscopic examination and resection of a polyp at the upper aspect of the duodenal bulb, especially adjacent to the pylorus, are difficult because of scope maneuverability.

After *Helicobacter pylori* eradication, a 73-year-old woman received a follow-up endoscopy, during which a duodenal polyp at the bulb was identified. A biopsy revealed gastric-type adenoma, and she was subsequently referred to our institute for further examination.

The polyp was located at the upper side of the bulb (Fig. 1). The oral side of the tumor was adjacent to the pylorus, but the anal margin could not be identified because of scope maneuverability, as the tumor was located at the upper aspect of the duodenal bulb. Despite using various types of endoscopes, including an ultrathin endoscope (GIF-H290Z, GIF-Q260J, and GIF-XP290N; Olympus, Tokyo, Japan), retroflex observation was impossible because of the narrow space of the bulb and the volume of the polyp itself. Noting no signs of invasion, such as submucosal tumor-like appearance,^1^ we elected to attempt endoscopic resection (Video 1, available online at www.videogie.org).

**Figure 1:**
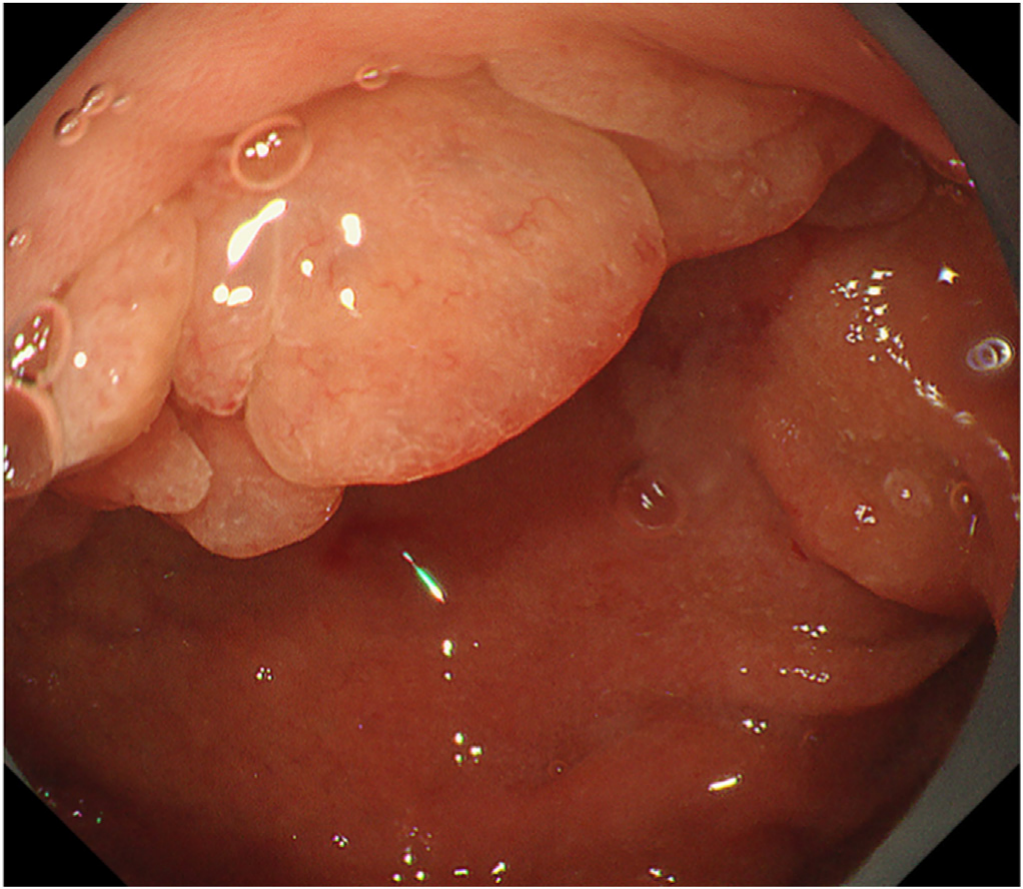
A duodenal polyp at the upper side of the bulb.

We prepared a newly developed thin endoscope (EG-840TP; Fujifilm, Tokyo, Japan) at 7.9 mm wide with a large working channel of 3.2 mm and wide angles (up 210° and down 160° each). However, retroflex observation at the bulb remained impossible and identifying the anal margin was difficult (Fig. 2). Underwater EMR was considered but dismissed because the polyp could not be snared with its location at the upper aspect of the duodenal bulb. Finally, we attempted endoscopic submucosal dissection (ESD) with the patient under intravenous anesthesia.

**Figure 2:**
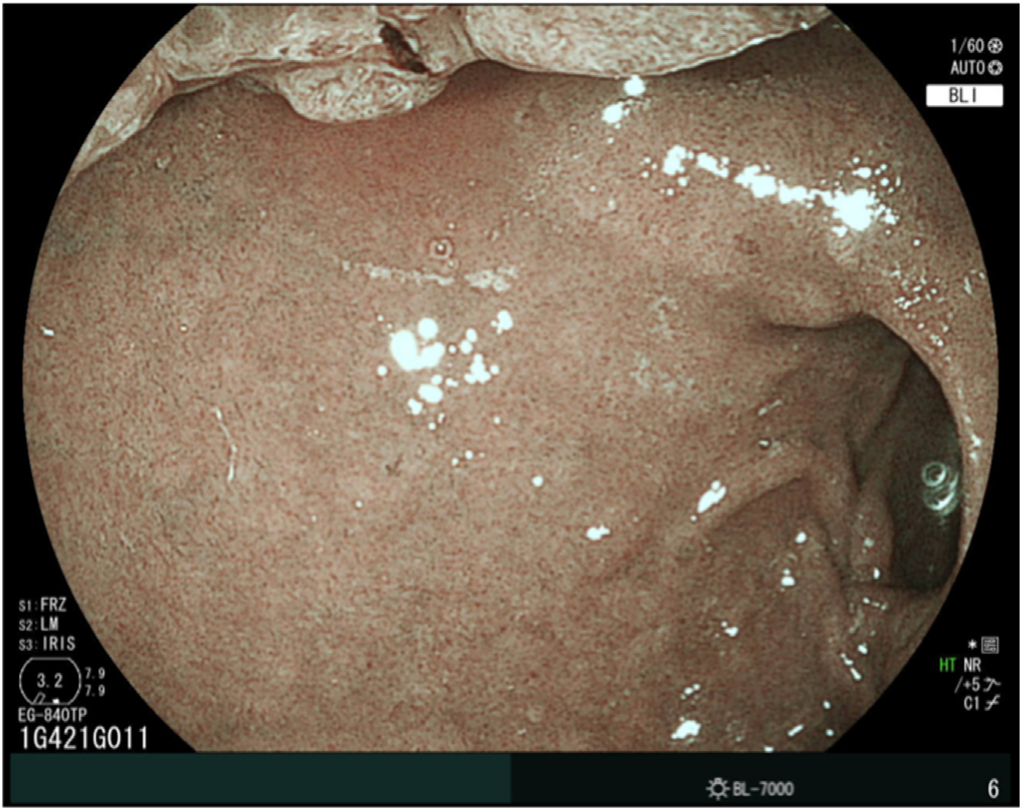
Identification of the polyp anal margin was difficult.

After injecting hyaluronic acid, mucosal incision began from the oral side (Fig. 3) with the endoscope attached with a hood (DH-083ST; Fujifilm). Using the pulley traction method (Fig. 3),^2^, ^3^, ^4^, ^5^ we proceeded with submucosal dissection from the oral side. The lesion subsequently repositioned to the anal side, leading to the identification of the anal margin and en bloc resection (Fig. 4). Microperforation occurred (Fig. 5) and was closed after resection (Fig. 6). Levofloxacin was administered to prevent peritonitis. The patient resumed her diet 2 days after ESD, and she was discharged on day 4.

**Figure 3:**
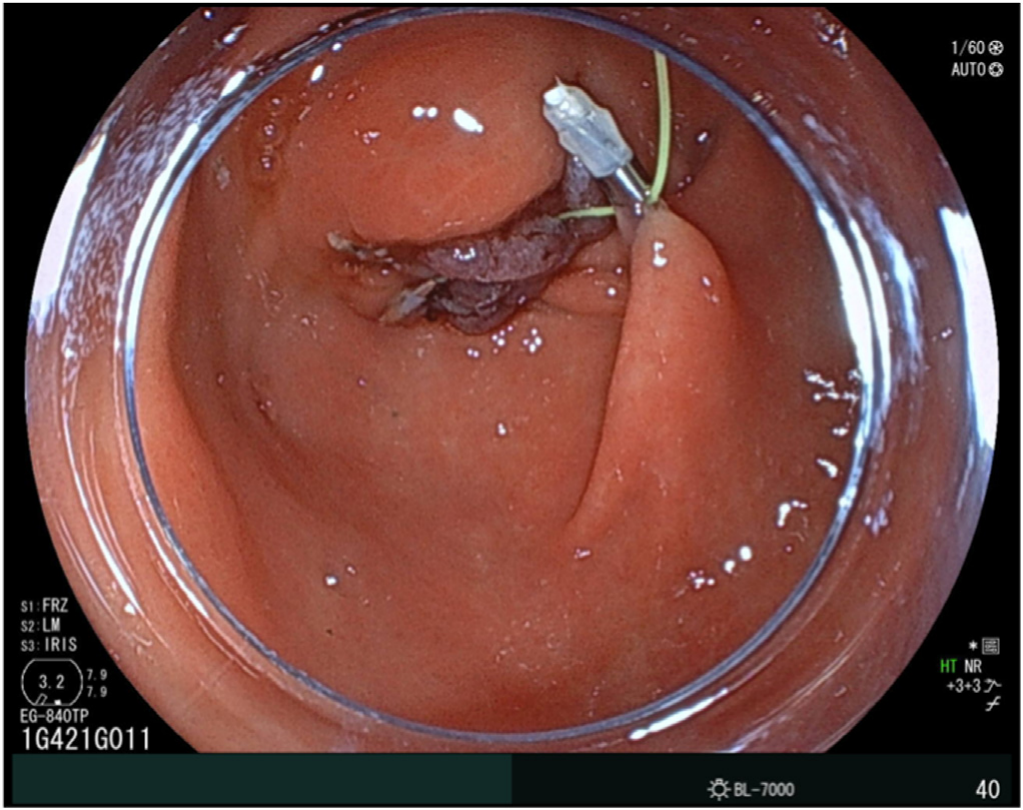
The pulley traction method was used to facilitate efficient submucosal dissection.

**Figure 4:**
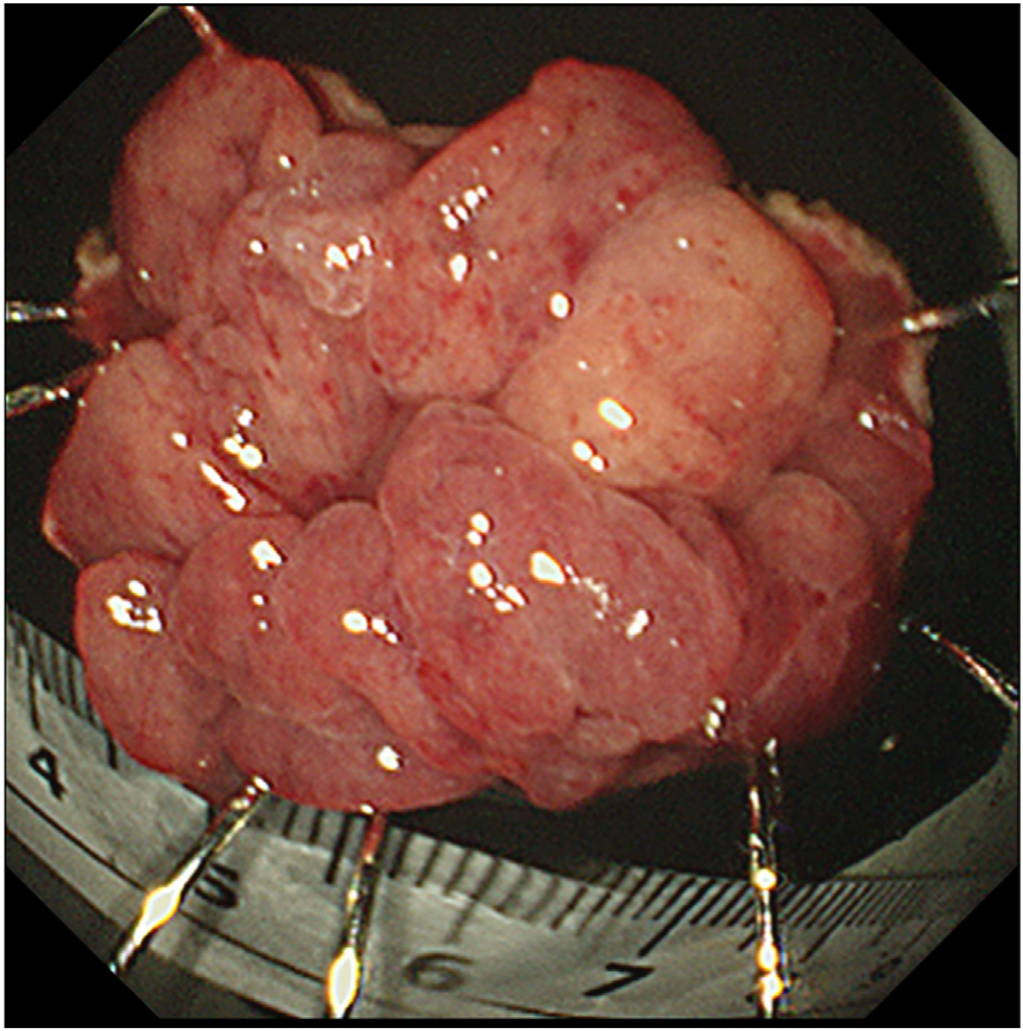
En bloc resection was achieved.

**Figure 5:**
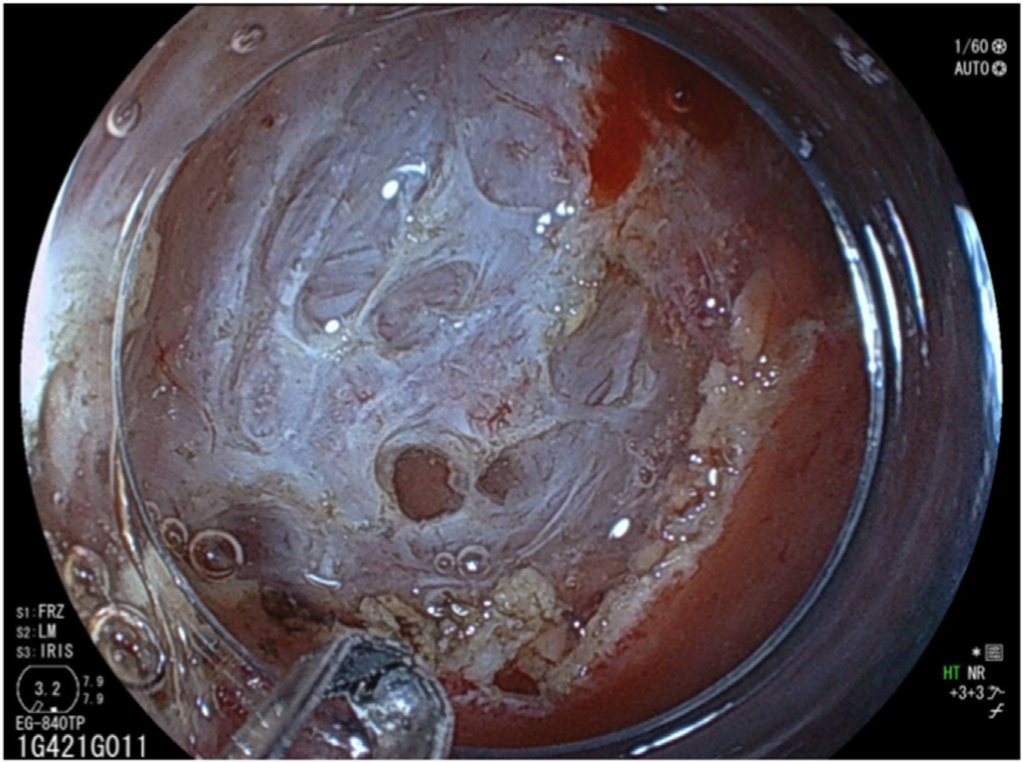
Microperforation after resection.

**Figure 6:**
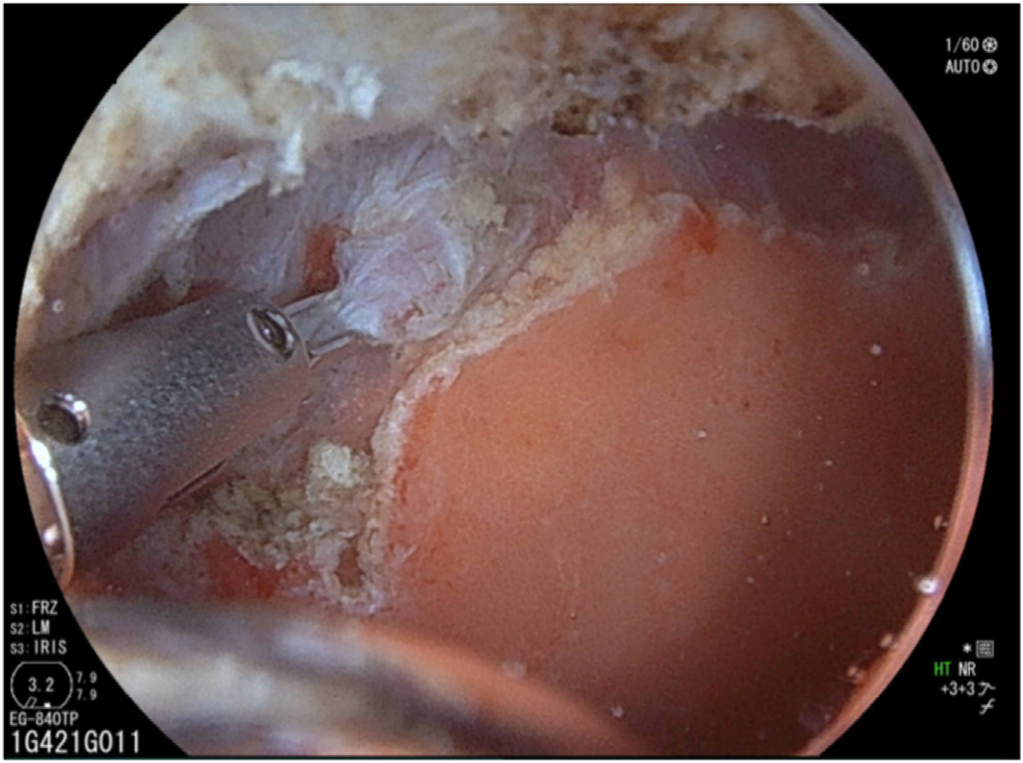
Microperforation was closed with a clip.

The final pathologic diagnosis was tubular adenoma, gastric type (immunostaining was focally positive with MUC5AC and positive with MUC6), with negative margins (Fig. 7). A follow-up endoscopy 2 months later confirmed no residual tumor (Fig. 8).

**Figure 7:**
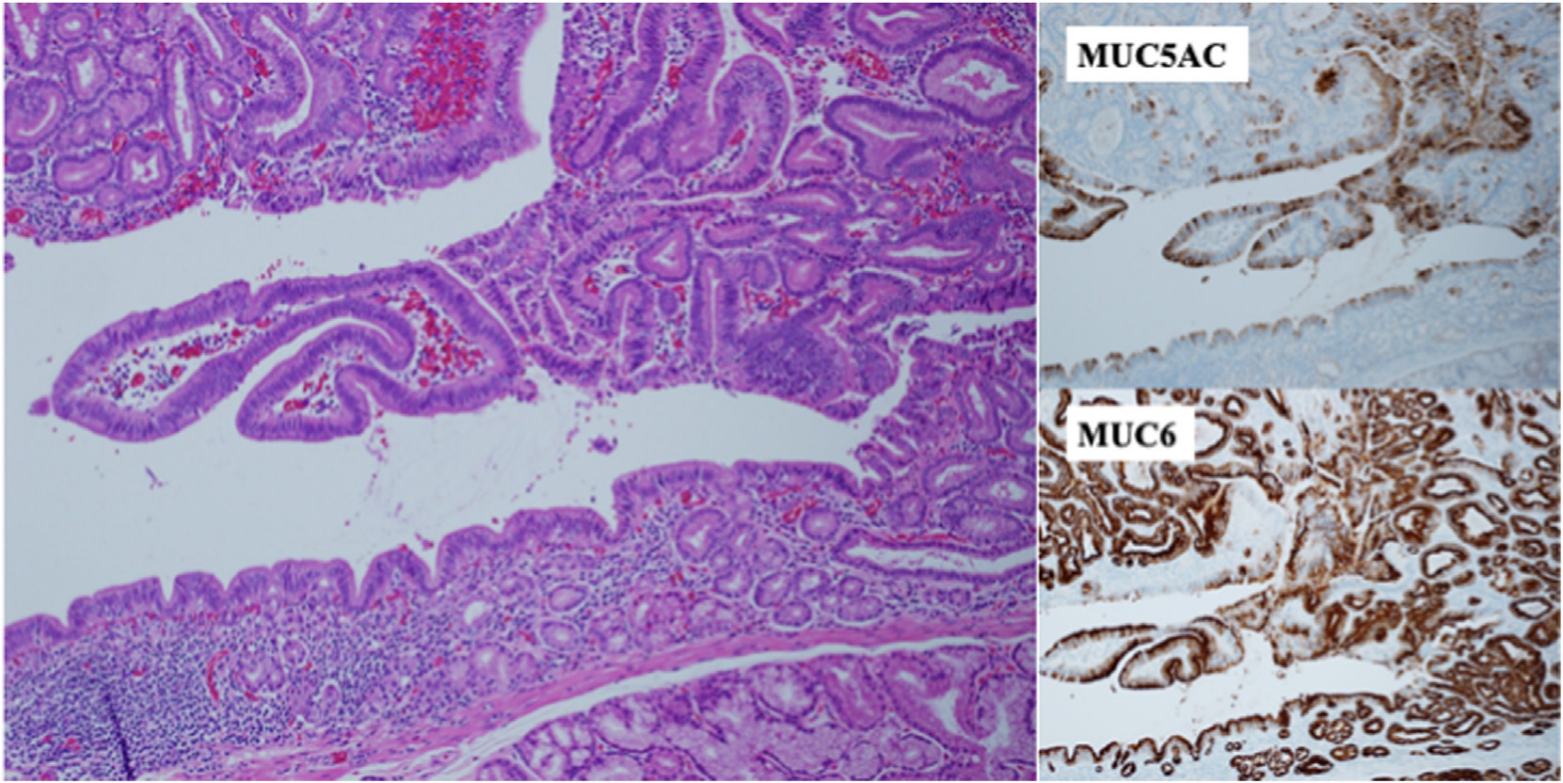
The final pathologic diagnosis was tubular adenoma, gastric type (immunostaining was focally positive with MUC5AC and positive with MUC6), with negative margins (H&E, immunostaining with MUC5AC and MUC6, respectively, orig. mag. ×100).

**Figure 8:**
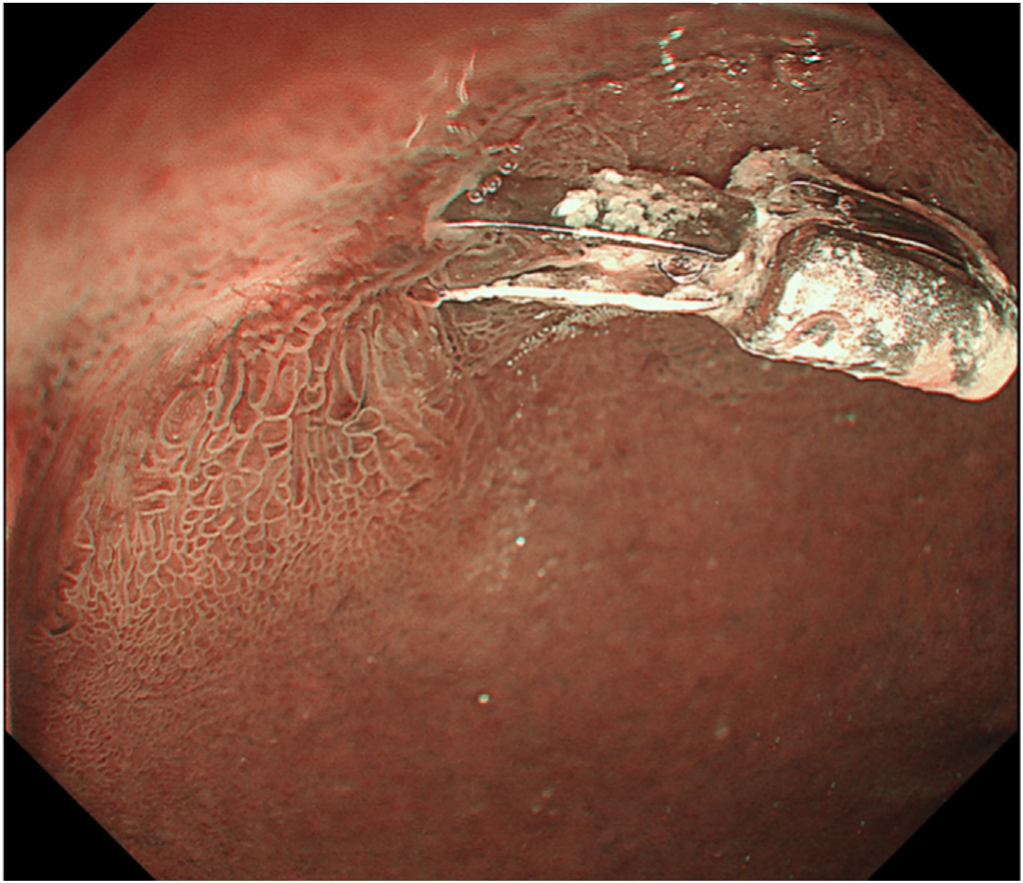
Narrow-band imaging after 2 months confirmed no residual tumor.

The new endoscope, with its small width at 7.9 mm and wide angles (up 210° and down 160° each) is especially useful for ESD with stricture or for a narrow space like a bulb or pharynx.

## Disclosure

The authors did not disclose any financial relationships.

## Supplementary data

Video 1 Endoscopic submucosal dissection for a duodenal polyp at the upper aspect of the duodenal bulb using a newly developed endoscope.

## References

[1] Takinami M., Kakushima N., Yoshida M. (2020). Endoscopic features of submucosal invasive non-ampullary duodenal carcinomas. J Gastroenterol Hepatol.

[2] Oyama T. (2012). Counter traction makes endoscopic submucosal dissection easier. Clin Endosc.

[3] Shichijo S., Matsuno K., Takeuchi Y. (2018). Pulley traction-assisted colonic endoscopic submucosal dissection affords good visibility of submucosal layer. VideoGIE.

[4] Shichijo S., Takeuchi Y., Matsuno K. (2019). Pulley traction-assisted colonic endoscopic submucosal dissection: a retrospective case series. Dig Dis.

[5] Shichijo S., Takeuchi Y., Waki K. (2020). Pulley traction-assisted endoscopic submucosal dissection with hemostatic forceps for a laterally spreading tumor in the ascending colon. VideoGIE.

